# Peripheral blood-derived mesenchymal stem cells demonstrate immunomodulatory potential for therapeutic use in horses

**DOI:** 10.1371/journal.pone.0212642

**Published:** 2019-03-14

**Authors:** Ana Leda F. Longhini, Tatiana E. Salazar, Cristiano Vieira, Thao Trinh, Yaqian Duan, Louise M. Pay, Sergio Li Calzi, Megan Losh, Nancy A. Johnston, Huisheng Xie, Minsu Kim, Robert J. Hunt, Mervin C. Yoder, Domenico Santoro, Taralyn M. McCarrel, Maria B. Grant

**Affiliations:** 1 Department of Ophthalmology and Visual Sciences, University of Alabama at Birmingham, Birmingham, Alabama, United States of America; 2 Department of Ophthalmology, Indiana University School of Medicine, Indianapolis, IN, United States of America; 3 Office of Vice President for Research, Indiana University, School of Medicine IACUC, Indianapolis, Indiana, United States of America; 4 Laboratory Animal Resource Center, Indiana University, Indianapolis, Indiana, United States of America; 5 College of Veterinary Medicine, University of Florida, Gainesville, Florida, United States of America; 6 Department of Clinical Science, College of Veterinary Medicine and Research Institute for Veterinary Science, Seoul National University, Seoul, Korea; 7 Hagyard Equine Medical Institute, Lexington, Kentucky, United States of America; 8 Department of Pediatrics, Indiana University School of Medicine, Indianapolis, Indiana, United States of America; Cedars-Sinai Medical Center, UNITED STATES

## Abstract

Previously, we showed that mesenchymal stem cells (MSC) can be mobilized into peripheral blood using electroacupuncture (EA) at acupoints, LI-4, LI-11, GV-14, and GV-20. The purpose of this study was to determine whether EA-mobilized MSC could be harvested and expanded *in vitro* to be used as an autologous cell therapy in horses. Peripheral blood mononuclear cells (PBMC) isolated from young and aged lame horses (n = 29) showed a marked enrichment for MSCs. MSC were expanded *in vitro* (n = 25) and administered intravenously at a dose of 50 x 10^6^ (n = 24). Treatment resulted in significant improvement in lameness as assessed by the American Association of Equine Practitioners (AAEP) lameness scale (n = 23). MSCs exhibited immunomodulatory function by inhibition of lymphocyte proliferation and induction of IL-10. Intradermal testing showed no immediate or delayed immune reactions to MSC (1 x 10^6^ to 1 x 10^4^). In this study, we demonstrated an efficient, safe and reproducible method to mobilize and expand, *in vitro*, MSCs in sufficiently high concentrations for therapeutic administration. We confirm the immunomodulatory function of these cells *in vitro*. This non-pharmacological and non-surgical strategy for stem cell harvest has a broad range of biomedical applications and represents an improved clinically translatable and economical cell source for humans.

## Introduction

Mesenchymal stem cells (MSC) are predominately derived from adipose tissue (AT) and bone marrow (BM) and have beneficial effects in a variety of clinical conditions [1, 2] including pain relief, immune modulation and tissue regeneration [3]. Bone marrow-derived MSC (BM-MSC) have been shown to result in improved tissue repair in experimental models of equine tendinopathy as well as in naturally occurring disease [4–6]. Intraarticular injection of BM-MSC has also resulted in decreased lameness in horses with meniscal lesions in combination with surgery [7]. While BM and AT remain the most common sources of MSC, they are not without associated risks. While uncommon, severe and potentially fatal complications of sternal bone marrow aspiration have been reported and include cardiac puncture, pneumopericardium, and arrhythmia [8]. Surgical collection of AT results in an obvious cosmetic blemish. While reported, minimally invasive AT collection in the form of liposuction is not widely available. Importantly, the site of AT harvest can affect the number of MSC recovered [9]. Prior attempts to isolate MSC from equine blood without mobilization as a simple and minimally invasive source of MSC were unsuccessful [10].

Previously, we demonstrated that electro-acupuncture (EA) performed using the limb acupuncture sites LI-4 and LI-11, with GV-14 and GV-20 (humans; known as *Bai-hui* in rats, mice, and horses) increased functional connectivity between the anterior hypothalamus and the amygdala in rats and humans[11]. This activation of the sympathetic nervous system (SNS) resulted in the mobilization of MSC into the systemic circulation [11]. In human patients, the source of the MSC was found to be primarily adipose tissue, whereas in rodents and horses, the tissue sources were considered more heterogeneous. Pharmacological disinhibition of rat hypothalamus enhanced SNS activation and similarly resulted in a release of MSC into the circulation [11]. EA-mediated SNS activation was further supported by browning of white adipose tissue in rats [11]. We also showed that EA treatment of rats undergoing partial rupture of the Achilles tendon resulted in a reduction of mechanical hyperalgesia, an increase of serum interleukin-10 (IL-10) levels and improved tendon remodeling, effects blocked in propranolol-treated rodents. These results indicate that EA activated sensory ganglia and SNS centers to mediate the release of MSC that enhance tissue repair, increase anti-inflammatory cytokine production and provide pronounced analgesic relief [11].

The purpose of the current study was three-fold, to first determine whether EA mobilized MSC (EA-MSC) would serve as a useful, safe, and minimally invasive source of expandable and therapeutic MSC, second to identify possible mechanism(s) for their therapeutic efficacy, and finally to investigate the effects of culture media components on immunogenicity and MSC characteristics.

## Materials and methods

All experimental protocols involving the initial characterization of the equine MSCs following EA were approved by the University of Florida (Institutional Animal Care and Use Committee (IACUC) protocol #201207468). These experiments were conducted in accordance with the guidelines and regulations of the IACUC at the University of Florida. The intradermal testing studies were also performed under the University of Florida IACUC protocol #2018-010 and all methods were carried out in accordance with relevant guidelines and regulations of University of Florida for this component of the study. The mobilization, expansion and re-administration of the expanded MSC to the horses were conducted under the Indiana University IACUC #10902. All of these experimental methods were carried out in accordance with relevant guidelines and regulations of Indiana University.

Horses (n = 29) received electro-acupuncture at acupoints LI-4, LI-11, and GV-14 and *Bai-hui* (immune points). Each set of points was stimulated by electricity at a frequency of 20 Hz for 45 minutes using an electro-acupuncture instrument (JM-2A model, Wuxi Jiajian Medical Instrument, Inc., Wuxi, China). This frequency has previously been used and shown to be effective [12]. The time chosen for the study is within the norm for an acupuncture session. To avoid artifact due to intrinsic circadian rhythmicity of stem cell release, all procedures were started at 9:00 a.m. and 60 ml of blood was collected 2–4 hours later in ethylenediaminetetraacetic acid (EDTA) containing tubes. Blood was maintained at room temperature (RT—~25°C) for transport to the lab for MSC isolation.

### Mononuclear cell isolation from peripheral blood

Within 24 hours following collection, peripheral blood mononuclear cells (PBMC) were isolated using Ficoll-Paque Plus (GE Healthcare Bio-sciences, Pittsburgh, Pennsylvania) through density gradient separation. In brief, blood was diluted 1:1 volume ratio with phosphate buffer solution (PBS) containing 2% fetal bovine serum (FBS) (Thermo Scientific, Waltham, Massachusetts, USA). The mixed blood was layered on top of half volume of Ficoll-Paque Plus in a 50 ml conical tube and centrifuged for 30 min at 1065 x g, acceleration 5, brake 1, RT. The buffy coat was transferred into a new tube containing PBS supplemented with 2% FBS and 1 mM EDTA and centrifuged again at 450 x g, RT for 10 min. Cell pellets were resuspended in 2 ml ammonium chloride (Stem Cell Technologies, Vancouver, Canada) and incubated at 4°C for 15 min to lyse red blood cells. Then the mixture was further washed three times with PBS containing 2% FBS. Cells were plated in EGM2/MEM medium [13] with 15% FBS at a density of 10^7^ cells/cm^2^ in T75 tissue culture flasks and allowed to attach for 4 days before the media was changed.

### Expansion and cryogenic preservation of EA-MSC

EA-MSC formed colonies which were expanded in T75 uncoated plastic flasks in 1:1 mix of EBM-2 (Lonza, Walkersville, Maryland, USA) and low-glucose MEMalpha (Lonza) medium and, 1% antibiotics/antimycotics; hereafter this is referred to as basal medium, and supplemented with 15% FBS. Media was exchanged every 2–3 days. Cells were passaged when cultures reached 80% confluence. Cells were detached from culture flasks using 0.25% Trypsin-EDTA solution, (Thermo Scientific, Massachusetts), washed once in basal medium, resuspended at a concentration of 5 x 10^6^ cells/mL in a mixture of 60% basal medium supplemented with 30% FBS and 10% dimethyl sulfoxide (DMSO, Sigma Aldrich, Carlsbad, California) and then slowly cooled to -80°C. Cells were then moved to liquid nitrogen for cryopreservation until administered.

### Phenotypic characterization of EA-MSC

To access the MSC phenotype, as proposed by the International Society for Cell Therapy, flow cytometry was performed to confirm expression of CD105, CD73 and CD90 [14]. Cells were washed in PBS containing 2% FBS and 1% of EDTA (staining buffer) and then resuspended in 100 μL of staining buffer. Antibodies were added in 4 different tubes: CD105-PE (Invitrogen, cat# MA-1-80944), CD146-Alexa Fluor 647 (Bio-Rad, cat# MCA2141A647), CD73-PE-Cy7 (BD Pharmingen, cat# 561258) and CD90-PerCP-Cy5.5 (BD Pharmingen, cat# 561557). The tubes were incubated for 20 minutes at RT, washed twice with 2 mL of staining buffer and analyzed by flow cytometry (BD Celesta, CA). The data was evaluated using FlowJo software (Tree Star, OR).

### Infusion of EA-MSC into horses

Autologous EA-MSC (n = 24) were shipped on dry ice to the veterinary facility. For each horse, five aliquots of 10x10^6^ cells in 1 mL of freezing medium (90% of FBS + 10% of DMSO) were thawed in a water bath at 37°C. Cells were resuspended in 50 mL of sterile PBS without calcium and magnesium (Thermo Scientific) (1% DMSO). The solution containing the cells was infused intravenously into the jugular vein. Horses were monitored immediately after infusion to detect any adverse effects. Five uninjected horses served as a control group. The horses’ owners monitored the horses for twelve weeks to determine if there were any changes in the horse’s general health.

In all cases the study horses had lameness due to osteoarthritis and had previously, unsuccessfully, been placed on oral supplements; Adequan (Luitpold Pharmaceuticals, Inc, Shirley, NY) or Legend (Merial, Inc., Duluth, GA). All treatments were discontinued at least one month prior to the initial acupuncture procedure used to harvest the MSC and horses were not on any medications during the subsequent 12 weeks of the study. The American Association of Equine Practitioners (AAEP)’s lameness scale was used as a standard for grading lameness before treatment and 6 weeks after EA-MSC infusion [15]. Grading of the horse initially and after 6 weeks was done by the study veterinarians.

### Co-culture of EA-MSCs and PBMCs for proliferation assay and IL-10 quantification

Blood was collected from 4 horses from which EA-MSC had already been isolated, and cells expanded as follows. To expand the cells, cell stocks were removed from liquid nitrogen and rapidly thawed in a 37°C water bath, washed with basal medium and plated on uncoated T75 flasks. Cells were passaged 3 to 5 times in basal media. For collection, cells were detached from culture flasks using 0.25% Trypsin-EDTA solution, and then washed five times in PBS supplemented with 2% horse serum. Cell pellets were resuspended in MEMalpha media and filtered through 40 μm strainers. Cells were then frozen as described above at 5 x 10^6^ cells/ml, except that freeze media consisted of 90% horse serum and 10% DMSO.

PBMCs were isolated and stained with Cell Trace (Thermo Fisher) following manufacturer’s recommendations and plated in 96-well flat plates that had been pre-coated 24 hours earlier with autologous EA-MSC. The MSC were plated at a concentration of 1x10^4^ MSC/well in 200 μL of DMEM supplemented with 20% of FBS. At the time of adding the PBMCs, 100 μL of medium was removed, and 100 μL of DMEM/ 20% FBS containing 1x10^5^ cells were added. Cells were cultured either with 2 μg/mL of phytohemmaglutinin-M (PHA) (Sigma) or no PHA at 37°C in 5% CO_2_ for 72 hours and 96 hours before the supernatants were aspirated and stored at -20°C for later analysis of IL-10 levels. As controls, MSC and PBMC were plated alone with or without PHA. The cells were harvested and analyzed by flow cytometry (BD Celesta). Analysis of lymphocyte proliferation was performed using ModFIT software (Verity Software). IL-10 was quantified according to manufacturer instructions (IL-10 Equine Elisa kit, Thermo Fisher).

### Intradermal testing

Eight horses, previously injected with EA-MSC that had been isolated, expanded, and frozen exclusively in media supplemented with FBS, were tested for immediate and delayed hypersensitive reaction via intradermal testing. The cohort of horses (age, number, and dates of injections) are described in S2 Table. Most horses received two intravenous injections. One horse received a single injection, whereas another horse received five injections. For each horse, the last injection was 13 to 24 months prior to the date of intradermal testing (IDT). Horses were determined to be systemically healthy before IDT based on a full physical examination and vital parameters (temperature, pulse, and respiration rate). Nine intradermal injections (0.15 ml) were administered on the left lateral neck and reactions were monitored at 0.33, 4, 24, 48, and 72 hours post-administration [16]. The injection site (10 x 15 cm) was clipped, and a permanent marker (Super Sharpie) was used to mark the injection sites. The test injections included: 0.9% sterile saline solution (negative control); 1:10,000 w/v histamine (positive control—immediate hypersensitivity reaction); 1 mg/ml phytohemagglutinin in sterile saline (positive control—delayed hypersensitivity reaction–T-cell mediated immune response) [17, 18]; 1x10^6^ EA-MSC; 5x10^5^ EA-MSC; 1x10^5^ EA-MSC; 5x10^4^ EA-MSC; and 1x10^4^ EA-MSC. The EA-MSC were prepared as described in the *Infusion of EA-MSC into Horses section* and suspended in sterile saline. All the injections were administered and the reactions evaluated in a subjective and objective manner by a board-certified veterinary dermatologist (DS). The subjective evaluation was applied only to the 20-minute readings, and it was based on the degree of turgidity, erythema, and size of swelling. The reactions were graded on a scale from 0 to 4 using the sterile saline as 0 and histamine as 4. The objective evaluation was based on the diameter in centimeters of each reaction.

### Expansion of EA-MSC in alternative mediums

At passage 3, the EA-MSC cultured in basal medium were passage in four different mediums: (1) Basal medium with 15% of FBS; (2) Basal medium with 15% of platelet lysate derived from peripheral blood of each horse (platelet lysate prepared as described by *Seo et al*. [19]); (3) Basal medium with 20% of heated autologous plasma and; (4) low-glucose MEMalpha (Lonza) with equine recombinant factors (4 x 10^−5^% of Equine FGF (250 μg/mL), R&D systems; 8 x10^-5^% of IGF-1 (250 μg/mL), Kingfisher Biotech Inc.; 2 x 10^−5^% of Equine VEGF-A (25 μg/mL), Innovative Research), 1 x 10^−3^% of hydrocortisone (20 μg/mL), 1.2% of heparin 2 mg/mL (Stem Cell) and 10 x10^-5^% of ascorbic acid (10 mg/mL) (Sigma). Hereafter, this is referred to as Horse Recombinant Medium (HRM), added 15% of autologous heat-inactivated horse serum. Four days later the cells were harvested, counted and analyzed by flow cytometry, as described above.

### Statistical analysis

Continuous data (dermal wheal area, IL-10 concentration) were tested for normality using the Kolmogorov-Smirnov test (alpha = 0.05). All variables were expressed as mean ± standard error of the means. Wilcoxon signed-rank test was performed for not-continuous data (lameness scores–S1 Table). For the *in vitro* studies, the data variables (IL-10, flow cytometry analysis) were analyzed by Student’s t-test. Friedman’s test was performed to evaluate the behavior of each data variable (continuous not normally distributed and ordinal [scores]) over time and among each of the solutions tested. If statistically significant (p values of ≤ 0.05), a Dunn’s Multiple Comparison Test was performed as post-hoc analysis. For the IDT, each reaction was compared to the 20-minute reaction (baseline) or to saline (negative control). To assess the over-time effect of FBS (objective evaluation IDT), a multiple comparison ANOVA followed by the Tukey’s multiple comparison test as post-hoc analysis was performed. Statistical significance (p-value) was set at < 00.5. Statistical analysis was done using GraphPad Prism6 software (GraphPad Software, Inc., San Diego, CA, USA).

## Results

### EA-MSCs derived from peripheral blood fit the phenotypic profile of equine MSCs and ameliorate lameness in horses with osteoarthritis

**Fig 1:**
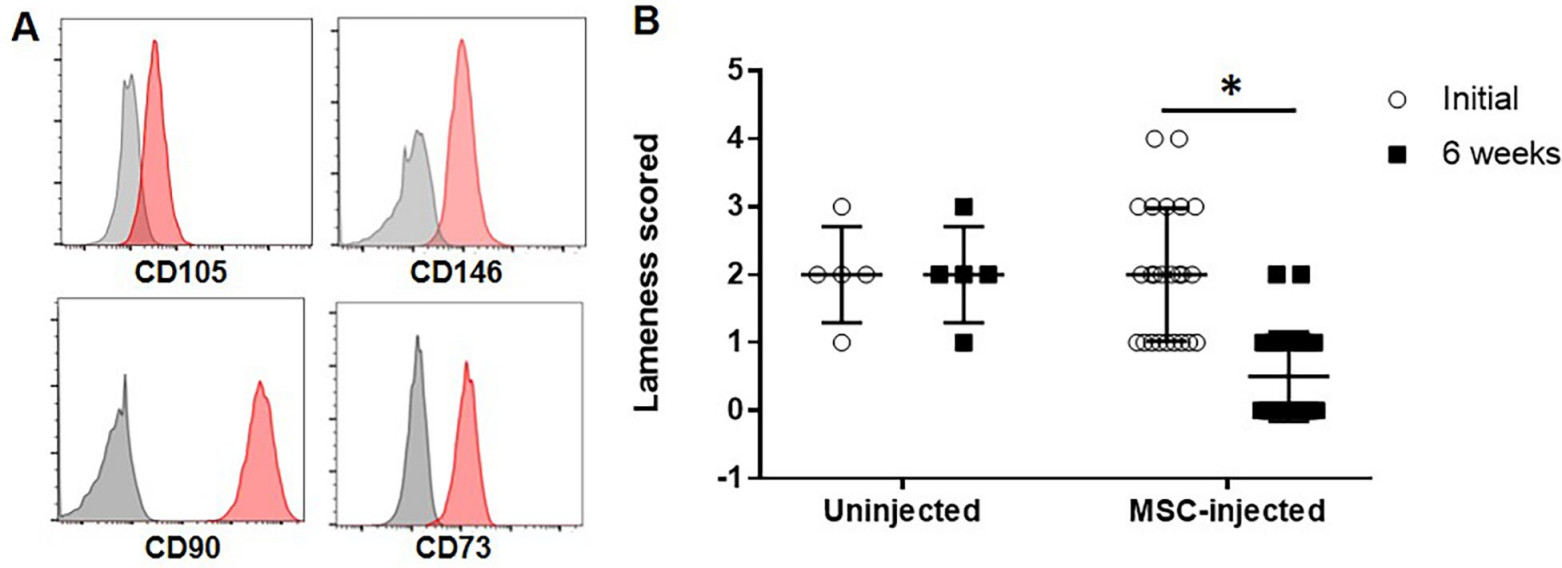
MSCs derived from horse peripheral blood expressed typical MSC markers and reduced lameness in horses with osteoarthritis. **(A)** Representative phenotype of MSC, gray line represents unstained cells; the red filled line represents cells stained with individual specific antibodies (CD105, CD146, CD90, CD73). **(B)** The AAEP lameness scale was used for grading lameness before and after six weeks. Autologous MSCs were injected intravenously in 24 horses and lameness was graded before and at 6 weeks following MSC infusion (p<0.0001, n = 24). No grading difference was observed between the initial evaluation and the 6-week assessment in horses not injected with MSCs (n = 5). Abbreviation: AAEP, American Association of Equine Practitioners. MSC, Mesenchymal stem cell.

Previously, we showed that EA-mobilized cells exhibit robust clonogenic potential and that the cells differentiated into osteocytes, adipocytes, and chondrocytes [11]. However, at the time of the original study, the cells were not characterized using flow cytometry due to the lack of availability of equine-specific antibodies. Now, with the availability of antibodies to CD105, CD146, CD90, and CD73, flow cytometry was performed to characterize the EA-MSC phenotypically. As shown in Fig 1A, the cells expressed CD 105, CD146, CD90 and CD73, typical markers of MSCs in passage 1.

Intravenous infusion of autologous EA-MSC (n = 24) did not result in any apparent adverse clinical responses following a single injection. The initial lameness and 6-week assessment is shown in Fig 1B and S1 Table. Mean age of horses was 12.9 ± 1.4 years. There was a significant improvement in lameness grade from a median pre-treatment grade of 2 (range 1–4) to a median post-treatment grade of 0 (range 0–2) (p<0.0001). Twenty-one of 24 horses improved by at least 1 grade 6 weeks after intravenous infusion of EA-MSC and 14 of 24 horses were assessed to have no observable lameness following treatment. Control horses (n = 5) did not show any clinical improvement (Fig 1B). A cohort of 7 horses were re-injected a second time or up to five additional times over a span of two years (S2 Table).

### EA-MSC have immunomodulatory effects *in vitro*

**Fig 2:**
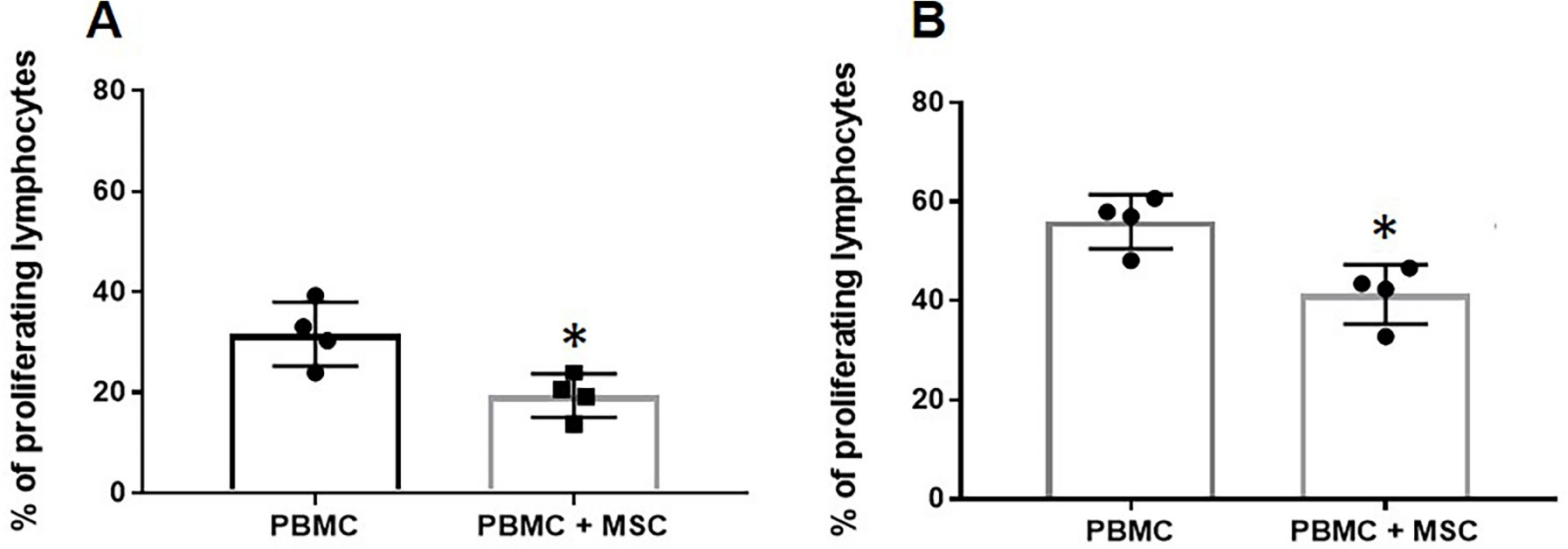
EA-MSC inhibiting lymphocyte proliferation. Cell tracer stained PBMC were stimulated *in vitro* with PHA (2μg/mL) in the presence or absence of autologous MSC in a ratio of 1 MSC: 10 PBMC for 72 hours **(A)** and 96 hours **(B).** Results are shown as Mean + SEM (n = 4). * indicates significant difference (p < 0.05). Abbreviations: PBMC, peripheral blood mononuclear cell; MSC, mesenchymal stem cell.

To investigate the beneficial effect of the EA-MSC infusion, the immunomodulatory effect of EA-MSC was examined. A decrease in proliferation was observed when PBMC were cultured in the presence of MSCs at 72 (Fig 2A) and 96 (Fig 2B) hours, suggesting EA-MSC are immunomodulatory and behave similarly to MSC derived from BM and AT [20].

**Fig 3:**
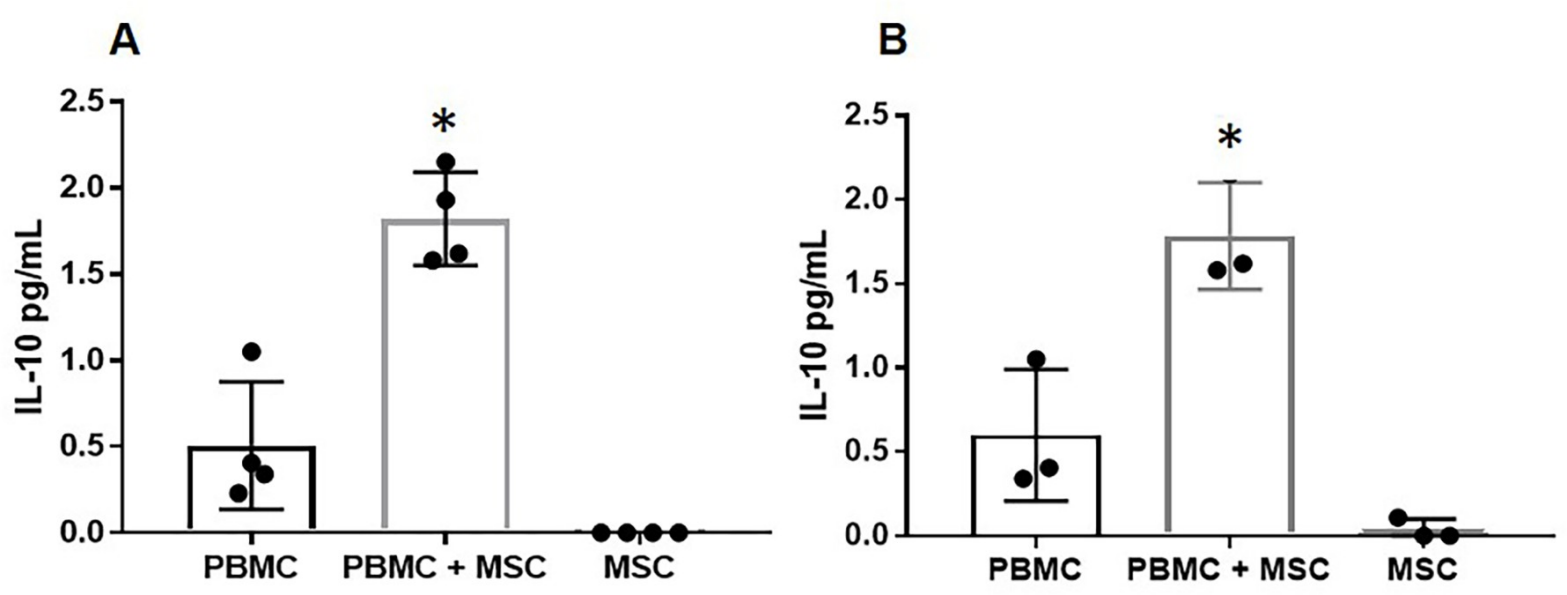
EA-MSC stimulate PBMCs to produce IL-10. PBMC were stimulated with PHA in the presence or absence of autologous MSC (1 MSC: 10 PBMC) for 72 hours **(A)** and 96 hours **(B)** and the IL-10 concentration was measured in the supernatant. Results are shown as Mean + SEM (*n* = 4). *indicates significant difference (*p* < 0.05). Abbreviations: PBMC, peripheral blood mononuclear cell; MSC, mesenchymal stem cell.

Further, at 72 (Fig 3A) and 96 hours, (Fig 3B), the medium collected from EA-MSC stimulated with PHA did not produce IL-10, although the equine PBMCs stimulated with PHA showed IL-10 production. However, when these cells were co-cultured with autologous EA-MSC, IL-10 production increased significantly. Unstimulated cells (PBMCs, EA-MSC, and co-cultured cells) did not release measurable amounts of IL-10 into media (data not shown).

### FBS induces both an immediate, late-phase, and delayed hypersensitivity reaction in horses with prior exposure

Repeated injections of EA-MSC could potentially provide benefit for treatment of chronic inflammatory conditions such as arthritis. However, repeated injection of foreign proteins, such as FBS used in the cell preparation, may result in a significant antigenic response. To determine whether FBS or other components of the infusion solution induced an antigenic response, IDT was performed. Autologous cells, while not expected to result in an immediate allergic reaction, were also used for IDT.

**Fig 4:**
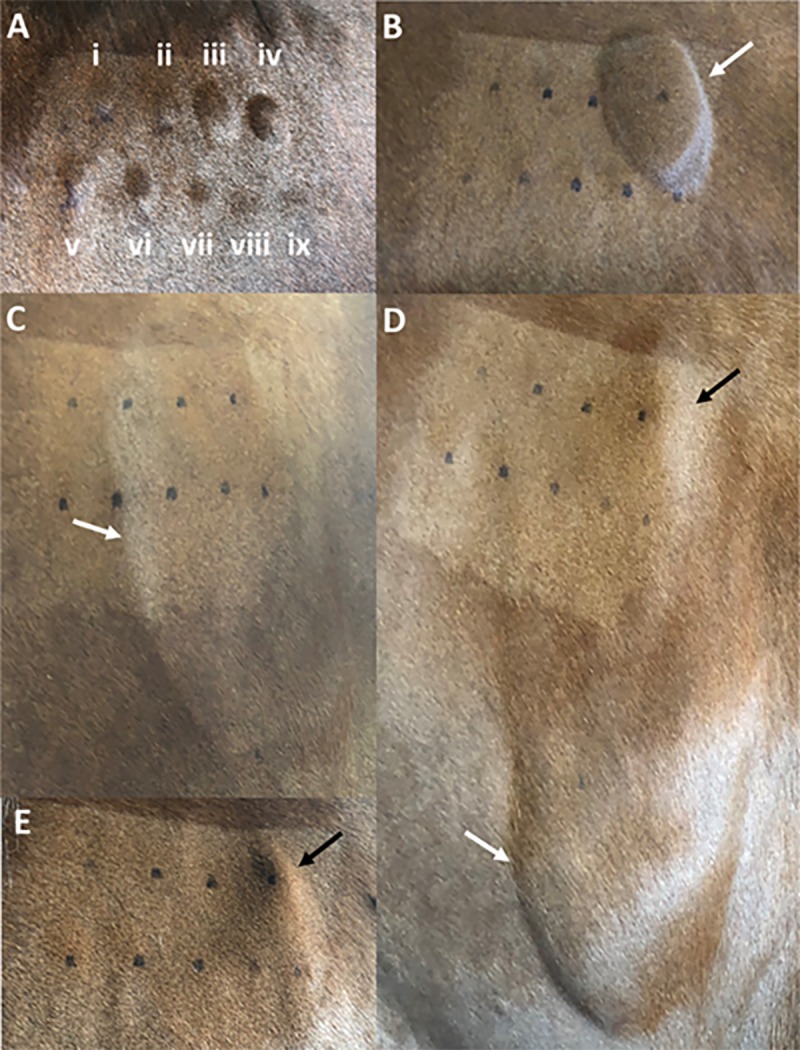
Intradermal testing. **A)** 20 minutes post-injection. Negative control (i), PHA (ii), histamine(iii), FBS (iv), and a linear decrease in reaction of the MSC (v to ix). **B)** Four hours post-injection shows the reaction elicited by the FBS. All the other injection sites were negative. **C)** Twenty-four hours post-injection shows an increase in the size of the FBS reaction with subcutaneous edema involving the lateral neck (black arrow). **D)** Forty-eight hours post-injection shows the increase in the size of the FBS reaction with subcutaneous edema starting from the injection site extending to the ventral lateral neck (dripping effect) (black arrow), and persistent localized swelling consistent with delayed hypersensitivity reaction (white arrow). **E)** Seventy-two hours post-injection showing the dramatic reduction in the subcutaneous edema and the persistent reaction due to the FBS (white arrow).

After 20 minutes, subjective evaluation of the intradermal injection of histamine and FBS demonstrated a significantly greater wheal formation compared to saline (p<0.0001 and p = 0.002, respectively). On the contrary, PHA and the EA-MSC were not associated with any significant induration. Reactions were recorded as positive if wheals were present at the later time points. PHA resulted in one positive reaction after 4 hours, and 24 hours, whereas two horses showed a positive reaction after 48 hours. No reactions were seen at 72 hours. Similarly, histamine elicited one positive reaction at 4 hours, two reactions at 24 hours, and one reaction at 48 hours. No reactions were seen at 72 hours. More interestingly, FBS elicited a strong positive reaction in all horses after 4 (late-phase) and 24 hours which remained as a positive reaction at 48 and 72 hours. As far as the EA-MSC concentrations tested, after 4 hours, two horses reacted to 1 x 10^6^ and 5 x 10^5^ dilution, whereas one horse had positive reactions to all dilutions. After 24 hours, a positive reaction was seen in two and one horse(s) for 1 x 10^6^ and 5 x 10^5^ dilution, respectively. After 48 and 72 hours, only one horse had a mild reaction to 1 x 10^6^ and 5 x 10^5^ dilution (Fig 4).

**Fig 5:**
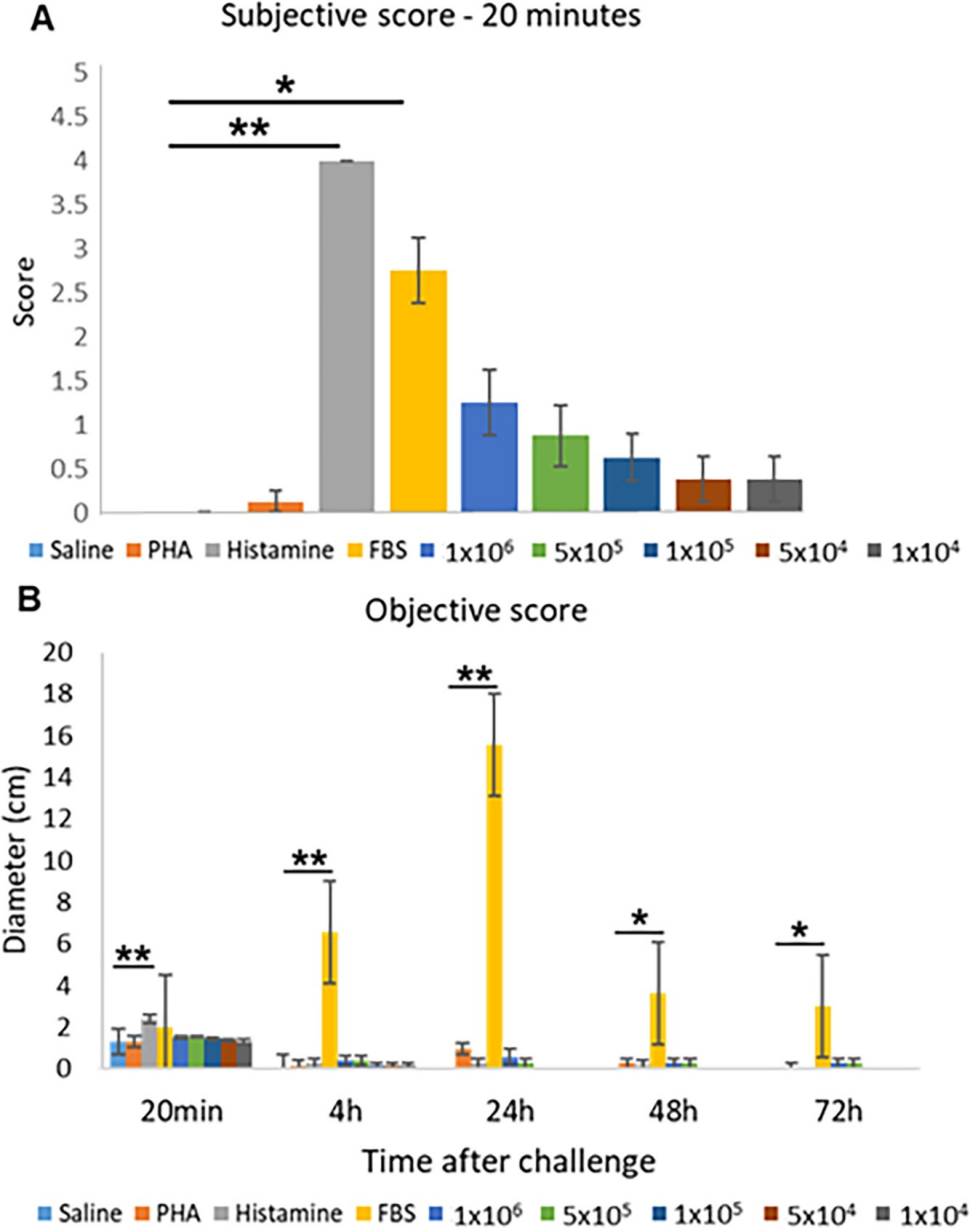
FBS induces an immediate and a delayed-type hypersensitivity reaction in previously exposed horses on intradermal testing. Eight horses were challenged with 5 different concentrations of autologous MSC, histamine, PHA, saline, and FBS. The acute reaction was measured at five different times after the initial challenge and is shown in cm. FBS caused a significant reaction at 4, 24, 48 and 72 hours. **(A)** Subjective score and **(B)** Objective score. Results are shown as means ± SEM (*n* = 8). * indicates significant difference (*p* < 0.05); ** indicates significant difference (*p* < 0.005). Abbreviations: MSC, mesenchymal stem cell; PHA: phytohemmagglutinin; FBS: fetal bovine serum.

The objective evaluation mirrored the subjective evaluation. After 20 minutes, the intradermal injection of histamine produced a significantly greater wheal formation compared to saline (p = 0.002). FBS consistently elicited a significantly higher response than saline (p≤0.02) for all time points. Surprisingly, PHA did not elicit a positive reaction at any time point. However, as expected, EA-MSC also did not stimulate any positive reaction (Fig 5).

### Autologous plasma or autologous platelet lysate are not effective replacements for FBS

**Fig 6:**
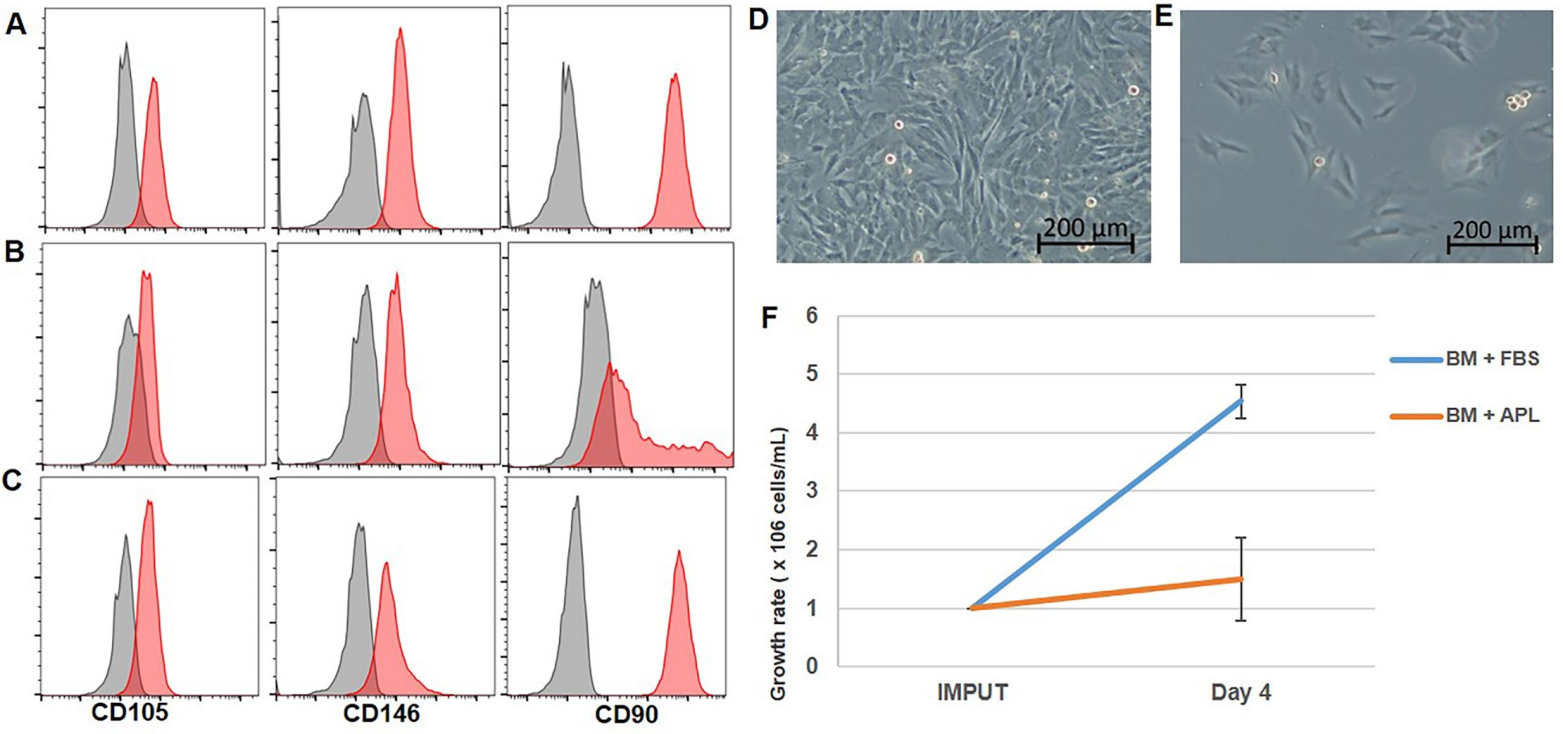
Basal medium with autologous plasma (AP) or autologous platelet lysate (APL) results in loss of cell surface marker phenotype and impaired growth respectively. Flow cytometry analysis shows lack of CD90 expression by MSC cultured for 4 days in basal medium + AP **(B)** compared to MSCs cultured in basal medium + FBS **(A)** and compared to MSCs cultured in basal medium + APL **(C)**. **(D):** MSC cultured for 4 days in basal medium + FBS, **(E):** MSC cultured for 4 days in basal medium + APL. **(F):** concentration of harvested cells was determined 4 days after the cells were plated and shows a decrease in the growth rate when the cells were cultured in basal medium + APL compared to cells cultured in basal medium + FBS (*n* = 2). Abbreviations: MSC, mesenchymal stem cell; FBS: fetal bovine serum.

Because exposure to FBS resulted in a strong antigenic response (immediate, late-phase, and delayed hypersensitivity reaction) and could lead to allergic reactions in horses following repeated MSC administration, we sought to develop a culture medium that was equally effective in promoting MSC expansion as the FBS containing medium. Basal media was a 50/50 mix of EBM-2 (Lonza), and MEMalpha (Lonza) supplemented with 15% of FBS. When EA-MSC were grown in basal medium supplemented with 15% autologous plasma, cells maintained a similar growth rate to EA-MSC exposed to FBS containing medium (data not shown). However, cell phenotype was affected as CD90 expression was lost (Fig 6C compared to Fig 6A). In contrast, cells grown in basal medium supplemented with autologous platelet lysate maintained expression of MSCs markers (Fig 6B); however, their growth rate was decreased by 50% compared with the cells cultured in basal medium containing FBS (Figs 6D and 5F).

### Horse recombinant medium supplemented with autologous serum maintained the growth rate and phenotype of EA-MSC

**Fig 7:**
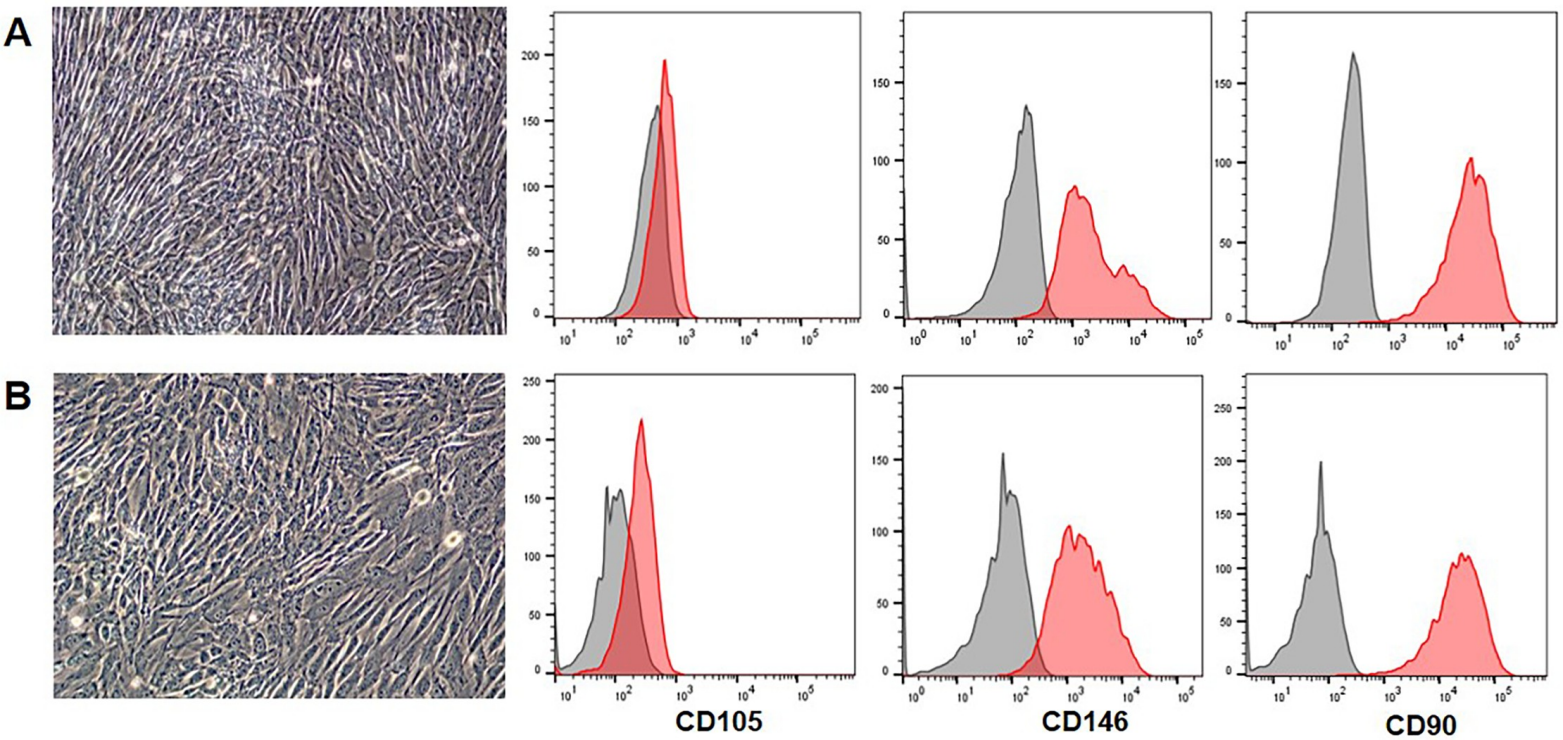
Horse recombinant media (HRM) supplemented with autologous serum (AS) exhibits similar growth promoting characteristics as basal medium supplemented with FBS. MSC cultured in basal medium + FBS **(A)** and HRM + AS **(B),** after 2 days of culture exhibit no difference in confluence or in the expression of mesenchymal markers.

To optimize the cell growth and avoid foreign proteins, the medium was prepared using equine recombinant growth factors, hydrocortisone, heparin and ascorbic acid (Horse Recombinant Medium [HRM]). HRM was supplemented with 15% of autologous horse serum and tested for EA-MSC expansion. EA-MSC cultures exposed to HRM +15% autologous horse serum showed similar growth rate and phenotype as EA-MSCs cultured in basal medium + 15% FBS (Fig 7). This supports the feasibility of using HRM +15% autologous horse serum for expansion of equine MSC and for minimizing allergic reactions with repeated administration. This was confirmed in two horses that received autologous MSC expanded with HRM+15% autologous serum and did not experience any adverse reaction.

## Discussion

This is the first report of peripheral blood-derived MSC for therapeutic acquisition without the use of drugs or growth factors for mobilization. EA successfully mobilized MSC into the bloodstream without adverse side effects such as fever, malaise, and pain which are often observed with other substances used for stem cell mobilization, for example, epinephrine, dopamine, substance P, GM-CSF or AMD3100 [21–24]. A single IV administration of autologous EA-MSC improved lameness in 21 of 24 horses treated, suggesting that EA-mobilized MSC represent a safe and efficacious option for therapeutic consideration. Advanced age did not appear to be a limitation to the mobilization and expansion of EA-MSC as these cells were isolated from elderly horses (S1 Table), and the cells maintained a robust growth rate in culture allowing clinical application in a population of horses most in need of treatment.

Autologous EA-MSC typically expanded to therapeutic concentrations in one month and were efficiently cryopreserved. Repeated administrations would be safe if cells were grown and cryopreserved using autologous serum supplemented with equine recombinant growth factors rather than FBS, as demonstrated by the IDT. We also compared various growth conditions to optimize expansion and preservation of MSC surface markers. Fukuda *et al*. showed that platelet-rich plasma was sufficient for growth of human BM-derived MSC [25]. We show that the use of equine platelet lysate was not a substitute for FBS as it resulted in a reduction in growth rate of EA-MSC. Human pre-clinical studies have supported the use of platelet lysate to avoid xenogeneic proteins. We observed that the addition of autologous plasma maintained the growth rate of EA-MSC, but affected the expression of classical MSC markers. In contrast, our study showed that use of equine recombinant growth factors and autologous heat-inactivated serum maintained cell growth and surface marker profile. In comparison to cells grown in FBS supplemented medium, the use of autologous and species-specific proteins should minimize the risk of antibody formation.

The beneficial effects of EA-MSC are likely mediated by immunomodulation, as we showed the cells were able to inhibit lymphocyte proliferation *in vitro* as has been demonstrated by equine MSC-derived from BM and fat [20]. This suggests that the equine MSCs induced regulatory T cell polarization. Luz-Crowford *et al*. demonstrated that BM-derived MSCs generate Tregs using stimulated CD4^+^ T cells in mice [26]. Equine MSCs have not only been shown to upregulate the production of T cell-modulating factors but also inhibit inflammatory mediator production such as interferon-gamma (IFN-γ) and tumor necrosis factor-alpha (TNF-α) and increase anti-inflammatory cell mediators such as transforming growth factor-beta (TGF-β) and prostaglandin E_2_ (PGE_2_) [27]. The TNF-α reduction is likely due to secretion of soluble TNF receptor II, which acts as an antagonist for TNF-α [28]. Importantly these findings have been confirmed in humans [29].

Kol *et al*. observed elevated splenic Foxp3^+^ Tregs in horses that received BM-MSC compared with horses that received AT-MSC and concluded that BM-MSC elevated CD8^+^ T-cells promoting development of Treg cells. Furthermore, the mechanism by which these MSCs of different origins decrease lymphocyte numbers *in vitro* also differs. AT-MSC induced T-cell apoptosis in activated T cells, whereas BM-MSC induced cell cycle arrest under similar conditions [15]. Furthermore, BM-MSC are more heterogeneous than AT-MSC suggesting that the source of MSC influences their immunomodulatory potential.

In our study, we also demonstrated that EA-MSC increase IL-10 production by PBMC. De White *et al*. showed that monocytes could produce IL-10 through phagocytosis of MSC [30]. However, we did not detect IL-10 in the supernatant of unstimulated co-cultured cells, indicating that the production of IL-10 in our study was most likely dependent on stimulated lymphocytes. MSC induced IL-10 production has been associated with trimethylation of histone H3K4me3 at the promoter of the FOXP3 gene locus, whereas it suppressed trimethylation of the corresponding region in the RORγ gene in Th17 cells. MSC mediate the adhesion of Th17 cells via CCR6 and exert anti-inflammatory effects through the induction of a T cell regulatory phenotype [31]. *In vitro*, MSC can also inhibit B-cell proliferation in both humans and horses [32, 33].

In this study, intradermal injections of different concentrations of EA-MSC did not elicit an immunological reaction, either acute nor delayed in the majority of horses. Since the intradermal route is a highly immunogenic route of administration, this test confirmed the safety of the EA-MSC. Interestingly, one horse that received only two intravenous injections of EA-MSC had mildly positive reactions at 4 hours’ post-intradermal injection, suggesting the possibility of a mild late-phase immune reaction in selected horses. In addition, it is noteworthy to mention that positive reactions to the highest concentrations of EA-MSC tested could be associated with a residual presence of FBS in the cell preparation. This was supported by one horse in which a clear correlation was seen between wheal size and EA-MSC concentration tested (Fig 4).

In summary, our study showed that EA allows for reproducible mobilization and harvesting of MSC from peripheral blood. This approach represents a minimally invasive and safe way to obtain MSC for therapy. The technique does not appear to be limited to young horses, and thus this strategy may offer an advantage over BM aspiration in older horses [34]. The immunomodulatory role of these cells included their ability to inhibit lymphocyte proliferation and stimulate PBMC to produce IL-10, which is likely due to an increase in T-regs. Significantly, this work provides a nonpharmacological and nonsurgical strategy for harvesting autologous cells in species that are able to undergo acupuncture.

## Supporting information

S1 Table Intravenous administration of MSCs was effective in producing improvement in the lameness grade in 21 of 24 horses with osteoarthritis. (DOCX)

S2 Table Demographic data and details on the intravenous injection administrations of EA-MSC in horses intradermally tested. (DOCX)

S1 Dataset (ZIP)
