# Supplementary material for: Peripheral blood-derived mesenchymal stem cells demonstrate immunomodulatory potential for therapeutic use in horses
Source: PLoS One. 2019 Mar 14;14(3):e0212642. doi: 10.1371/journal.pone.0212642 (PMC6417789; doi:10.1371/journal.pone.0212642)
Supplement: S1 Table — (DOCX) [file pone.0212642.s001.docx]

| **Lameness scored according to AAEP (F = mare, G = gelding)** | | | | |
| --- | --- | --- | --- | --- |
| **Horse** | **Age** | **Gender** | **Initial** | **Final** |
| **1** | 27 | G | 3 | 1 |
| **2** | 17 | G | 2 | 0 |
| **3** | 9 | F | 3 | 0 |
| **4** | 15 | F | 4 | 2 |
| **5** | 35 | G | 3 | 0 |
| **6** | 9 | G | 3 | 0 |
| **7** | 15 | G | 3 | 0 |
| **8** | 5 | G | 2 | 0 |
| **9** | 20 | F | 4 | 2 |
| **10** | 14 | F | 1 | 1 |
| **11** | 17 | F | 1 | 1 |
| **12** | 13 | F | 1 | 1 |
| **13** | 7 | G | 2 | 1 |
| **14** | 6 | F | 2 | 0 |
| **15** | 7 | F | 2 | 1 |
| **16** | 7 | G | 1 | 0 |
| **17** | 9 | G | 1 | 0 |
| **18** | 11 | G | 2 | 1 |
| **19** | 16 | G | 1 | 0 |
| **20** | 6 | F | 1 | 0 |
| **21** | 8 | G | 2 | 1 |
| **22** | 10 | G | 1 | 0 |
| **23** | 17 | G | 2 | 0 |
| **24** | 10 | G | 1 | 0 |
| **25** | 9 | G | 2 | 2 |
| **26** | 17 | F | 1 | 1 |
| **27** | 23 | G | 2 | 2 |
| **28** | 19 | G | 3 | 3 |
| **29** | 9 | G | 2 | 2 |
