# Supplementary material for: Peripheral blood-derived mesenchymal stem cells demonstrate immunomodulatory potential for therapeutic use in horses
Source: PLoS One. 2019 Mar 14;14(3):e0212642. doi: 10.1371/journal.pone.0212642 (PMC6417789; doi:10.1371/journal.pone.0212642)
Supplement: S2 Table — (DOCX) [file pone.0212642.s002.docx]

| **Horse** | **Age** | **# of**  **injections** | **Injection**  **date #1** | **Injection**  **date #2** | **Injection**  **date #3** | **Injection**  **date #4** | **Injection date #5** |
| --- | --- | --- | --- | --- | --- | --- | --- |
| #3 | 11 | 2 | 02/09/2016 | 04/15/2016 |  |  |  |
| #8 | 7 | 2 | 02/09/2016 | 04/15/2016 |  |  |  |
| #13 | 8 | 2 | 02/09/2016 | 04/15/2016 |  |  |  |
| #16 | 9 | 1 | 02/09/2016 |  |  |  |  |
| #18 | 14 | 2 | 06/15/2015 | 08/10/2016 |  |  |  |
| #19 | 18 | 2 | 06/15/2016 | 08/10/2016 |  |  |  |
| #25 | 5 | 2 | 02/09/2016 | 04/15/2016 |  |  |  |
| #26 | 13 | 5 | 06/15/2015 | 12/15/2015 | 06/20/2016 | 12/20/2016 | 06/15/2017 |

Two additional horses (#25 & #26) that received EA-MSC were not included in Table 1 as they did not receive their 6-week evaluation by the study veterinarian. Most of the horses enrolled were treated with two injections two months apart. Only one horse was treated with a single injection and another horse with five injections six months apart. No potential associations between the number of injection administered and the degree of the wheal size was seen.
